# Multiscale dynamics of interstimulus interval integration in visual cortex

**DOI:** 10.1371/journal.pone.0208822

**Published:** 2018-12-17

**Authors:** J. Alegre-Cortés, C. Soto-Sánchez, E. Fernandez

**Affiliations:** 1 Bioengineering Institute, Miguel Hernández University (UMH), Alicante, Spain; 2 Biomedical Research Networking center in Bioengineering, Biomaterials and Nanomedicine (CIBER-BBN), Madrid, Spain; 3 Biotechnology Department, University of Alicante (AU), Alicante, Spain; Georgia State University, UNITED STATES

## Abstract

Although the visual cortex receives information at multiple temporal patterns, much of the research in the field has focused only on intervals shorter than 1 second. Consequently, there is almost no information on what happens at longer temporal intervals. We have tried to address this question recording neuronal populations of the primary visual cortex during visual stimulation with repetitive grating stimuli and intervals ranging from 1 to 7 seconds. Our results showed that firing rate and response stability were dependent of interval duration. In addition, there were collective oscillations with different properties in response to changes in intervals duration. These results suggest that visual cortex could encode visual information at several time scales using oscillations at multiple frequencies.

## Introduction

Despite the increasing interest in how visual cortex neurons encode visual information [1–4], there are still many open questions concerning the temporal integration at long (seconds) scale. While the encoding of sensory information at sub seconds scale has been extensively studied [5–8], the processing of longer time intervals has traditionally been less studied[9–12], and it has usually been addressed via pharmacological studies [13]. An exception to this is the study of reward timing in visual cortex; during the last years it has been discovered that reward timing [10] and visually cued action timing [12] are represented in visual cortex in experiments on which the delay between the cue and the reward lasted up to 3 s.

How the brain encodes temporal information is indeed an open field of research [14–16]. Thereby, some models propose that time is encoded in populations of pacemaker neurons that are the only responsible of temporal coding in the brain via oscillating at different frequencies [17], while others propose that time is encoded by arrays of neurons with different time constants that respond to different intervals. Furthermore, there is evidence showing that *in-vitro* cortical networks can reflect specific temporal patterns [18].

On the other hand, many theories of perception are based on cortical oscillations [19] which have been well characterized in response to visual stimulation [20,21], attention [22], speech recognition [23], motor function [24] or reward expectation in visual cortex [25,26]. Furthermore, our sensory environment is full of regularities, for example, repetitive stimuli, which we use to predict future events. In this framework, the main goal of this study was to get insights about if neuronal populations of deep layers of visual cortex can convey and process interval information at seconds scale using spikes. To answer this question, we recorded neuronal populations from rat visual cortex while visually stimulated with a unique type of stimulus and different interstimulus intervals (ISI), ranging from 1 to 7 seconds.

To obtain a high-resolution picture of population oscillations we decomposed the signals obtained averaging all the electrodes using a Noise Assisted Multivariate Empirical Mode Decomposition (NA-MEMD) [27] and computed the Huang-Hilbert Spectrum (HHS) for nonlinear and non-stationary time series analysis. This procedure was chosen because it allows to analyze the recordings with instantaneous temporal and frequency resolutions [28–30], which is crucial to depict nonlinear properties of the responses [31–33] and moreover it is robust against signal intermittency in the data [27,34][35]. Our results showed that the responses of visual cortex neuronal populations to an unchanged grating stimulus had different Time-Frequency (T-F) dynamical spectra, depending on which ISI was used. Furthermore, these spectral differences were present at different frequencies and times which fully agrees with temporal multiplexing theory [36,37].

## Materials and methods

### Experimental design

Visual cortex multiunit recordings were obtained from Long Evans adult rats (n = 4, Janvier Labs, France) weighing 450–500 gr. Analgesia was induced by buprenorphine (0.025mg kg-1 s.c), and surgical anesthesia and sedation were induced by ketamine HCl (40 mg kg^-1^ i.p). The anesthesia was continued and maintained with a mix of oxygen and 2.5% of isoflurane during the surgery and afterward reduced to 1.5% during the electrophysiological recordings. The blinking and the toe pinch reflexes were continuously checked along the experiment to guarantee a proper level of anesthesia for the animal. The body temperature was maintained with a thermal pad and the heart rate and O_2_ concentration in blood were monitored throughout the experiment. Animals were pre-treated with dexamethasone (1 mg kg^-1^ i.p) 24 hours and 20 minutes prior to surgery in order to avoid brain edema caused by the electrode insertion.

A craniotomy was drilled on top of the visual cortex and a customized 6x6 Utah Electrode Array (UEA) covering a brain surface of 2 mm x 2 was inserted 2 mm lateral to the midline and 0.5 mm anterior to lambda. The UEA was inserted in the deep layers of the visual cortex (>600 μm) through the duramatter with the help of a Blackrock pneumatically-actuated inserter device (Blackrock Microsystems, Salt Lake City, USA). After the insertion, the ipsilateral eyelid to the craniotomy site was closed with cyanoacrylate and atropine sulphate 1% was used to dilate the pupil of the contralateral eye.

*In vivo* neural activity from visual cortex was recorded simultaneously from 16 individual electrodes. The UEA array was connected to a MPA32I amplifier (Multichannel Systems, MCS) and the extracellular recordings were digitized with an MCS analog-to-digital board. The data were sampled at a frequency of 20 kHz and slow waves were digitally filtered out (100–3000 Hz, 2^nd^ order Butterworth type IIR digital filter) from the raw data. Neural spike events were extracted with a free-tool application for offline spike sorting analysis (Neural Sorter, http://sourceforge.net/projects/neuralsorter/) and the resulting multiunit information obtained from each electrode was stored for further analysis.

**Fig 1:**
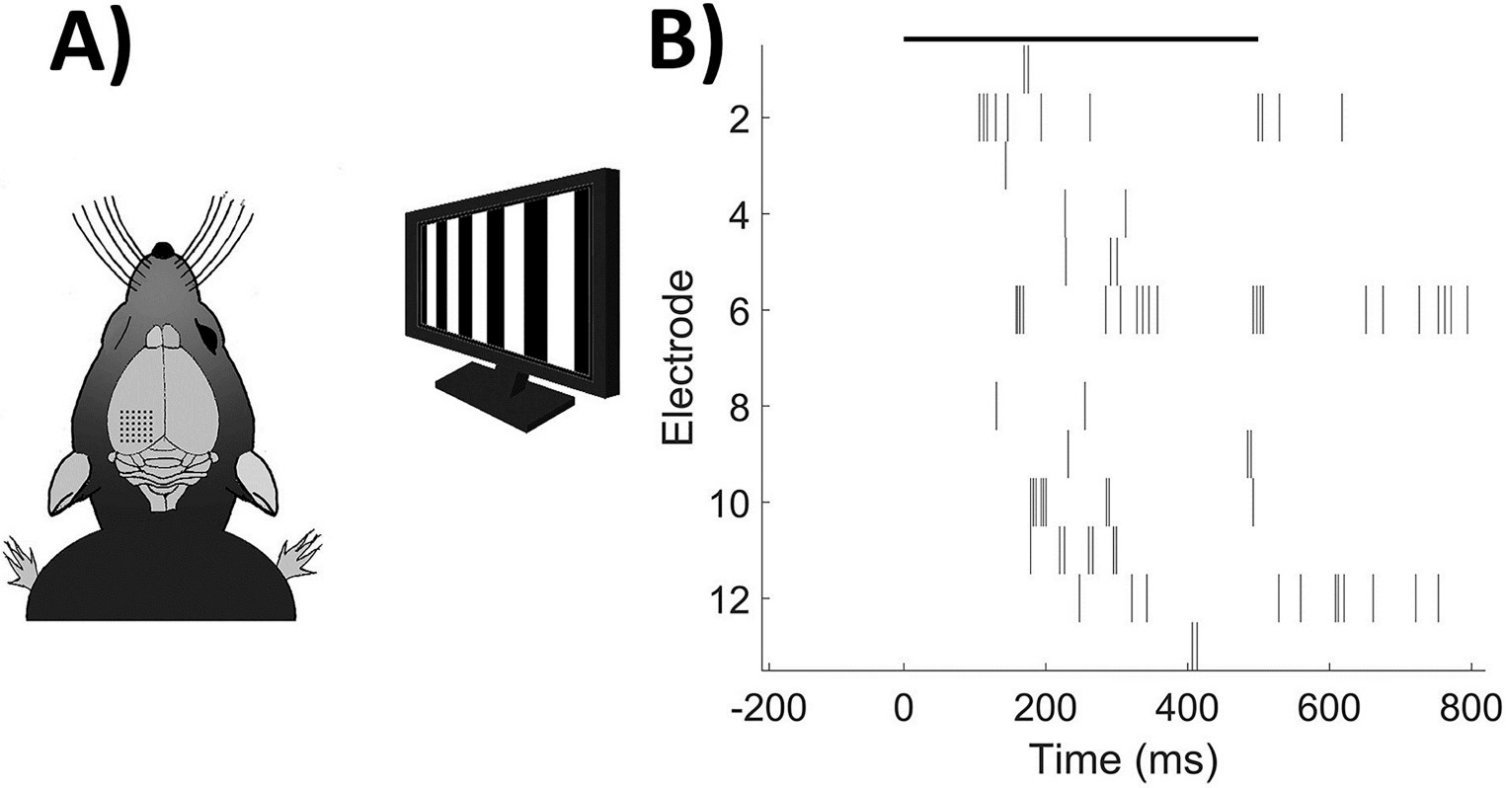
Experimental paradigm and representative trial. A) Schematic representation of the experimental stimulus-recording design. B) Example raster plot of a single trial and all electrodes. Stimulus displayed as black line.

Visual stimulation consisted on a vertical drifting square-wave grating (90°, light and dark bars, 100% contrast, 6 Hz, 0.6 cycles/degree) of 500 ms duration interspersed with a dark (uniform) stimulus of variable duration. The stimulus was displayed on an LCD monitor (refresh rate 60 Hz) and a luminance of ≈100 cd/m^2^, placed 25 cm in front of the right eye, approximately at 30° from the midline covering a visual field spanning of ≈100° (Fig 1A) and repeated 15 times for each ISI. We used 1, 3, 5 and 7 seconds ISI protocols in separated experiments. The stimuli were generated using the Vision Egg library and customized python scripts. The room was kept in darkness all along the visual stimulation experiments. Euthanasia of the animals was performed once the recording protocols were ended.

### Ethical approval

All the experimental procedures performed in this study conform to the directive 2010/63/EU of the European Parliament and of the Council, and the RD 53/2013 Spanish regulation on the protection of animals use for scientific purposes and were approved by the Miguel Hernandez University Committee for Animal use in Laboratory.

### Data analysis

Neural activity analysis was performed in Matlab (MathWorks). Single or multiunit spiking activity was isolated from the background. We observed multiunit activity in the majority of the electrodes through the whole recording sessions, then, only those electrodes with neural activity higher than 0.5 spikes/s were considered in the analysis. (Fig 1B).

We constructed time-dependent population activity vectors by temporally binding the activity of each electrode with 1 ms resolution. We considered one second before and one second after each stimulus presentation for each ISI. The first stimulus of each recording was not considered for the analysis. Possible anticipation dynamics were not subject to analysis, as they exceeded the aim of this paper.

### NA-MEMD

We used an extension of the EMD algorithm[38] to study the T-F properties of the neural response. It has recently described [30,39] that the result of using EMD family of algorithms to study the oscillatory properties of spike trains improves the results obtained by means of using other traditional T-F techniques due to the presence of nonlinearities and nonstationarities in the signal [30–32,40].

To overcome the problems associated to univariate EMD analysis, we used the Multivariate Empirical Mode Decomposition (MEMD) [41], which is a multivariate extension of EMD algorithm [38], where analysis of simultaneous dimensions is performed simultaneously to obtain a meaningful decomposition of the whole multidimensional signal in a subset of vectors called Intrinsic Mode Functions (IMFs). Furthermore, we added White Gaussian Noise (WGN) to the MEMD, which increases its performance via reducing mode mixing produced by signal intermittency [34]. This procedure acts as a filter bank that enhances time-frequency resolution [41, 42]. Briefly, WGN is added in additional dimensions:

d=n+k,

Where *d* is the final number of dimensions, *n* the dimensions of the original data (electrodes, trials…) and *k* the number of additional dimensions including WGN [41].

In order to apply this Noise Assisted Multivariate Empirical Mode Decomposition (NA-MEMD) to our data, we adapted the MEMD Matlab package (http://www.commsp.ee.ic.ac.uk/~mandic/research/emd.htm). We used the low-discrepancy Hammersley sequence to generate a set of 300 direction vectors for computing signal projections and 4 WGN channels. Standard stopping criterion was described elsewhere [43].

Since in our study, we seek for depicting features present in the whole recorded population, we averaged the activity of all electrodes for the presentations of the moving grating for each ISI in each animal and then added 4 additional dimensions with WGN.

### Hilbert transform

We measured present frequencies in our data as the instantaneous frequency (IF) using Hilbert Transform (HT) (Huang et al. 1998). For a given time series x(t), its Hilbert Transform H(x)(t) is defined as:

$$H(x)(t)=\frac{1}{\pi }C\int_{-\infty }^{\infty }\frac{x(t^{\prime })}{t-t\prime }dt\prime ,$$

Where C indicates the Cauchy principal value. Hilbert Transform results in a complex sequence with a real part which is the original data and an imaginary part which is a version of the original data with a 90° phase shift. This *analytic signal* is useful to calculate instantaneous amplitude and frequency, thereby instantaneous amplitude is the amplitude of H(x)(t), and the instantaneous frequency is the time rate of change of the instantaneous phase angle.

### Phase space

We created a low dimensional phase space considering mean population firing rate as well as two IMFs that statistically carried ISI information with different temporal dynamics and were consistent with multiplexed theory, IMF 8 (5.93 +/- 0.37 Hz) and IMF 6 (18.37 ± 1.29) [36,37].

To compare the Euclidean distance of the mean population trajectories in this phase space we normalized all axes to prevent from any biasing and computed the center of all trajectories. A 200 ms duration window of time ranging from 400 ms to 200 ms before stimulus onset was used to localize the fixed point representing the population resting state before stimulation.

### Statistical analysis

All experimental comparisons were tested using Wilcoxon rank-sum test. P values for multiple comparisons were corrected ad hoc using Storey method [44].

## Results

**Fig 2:**
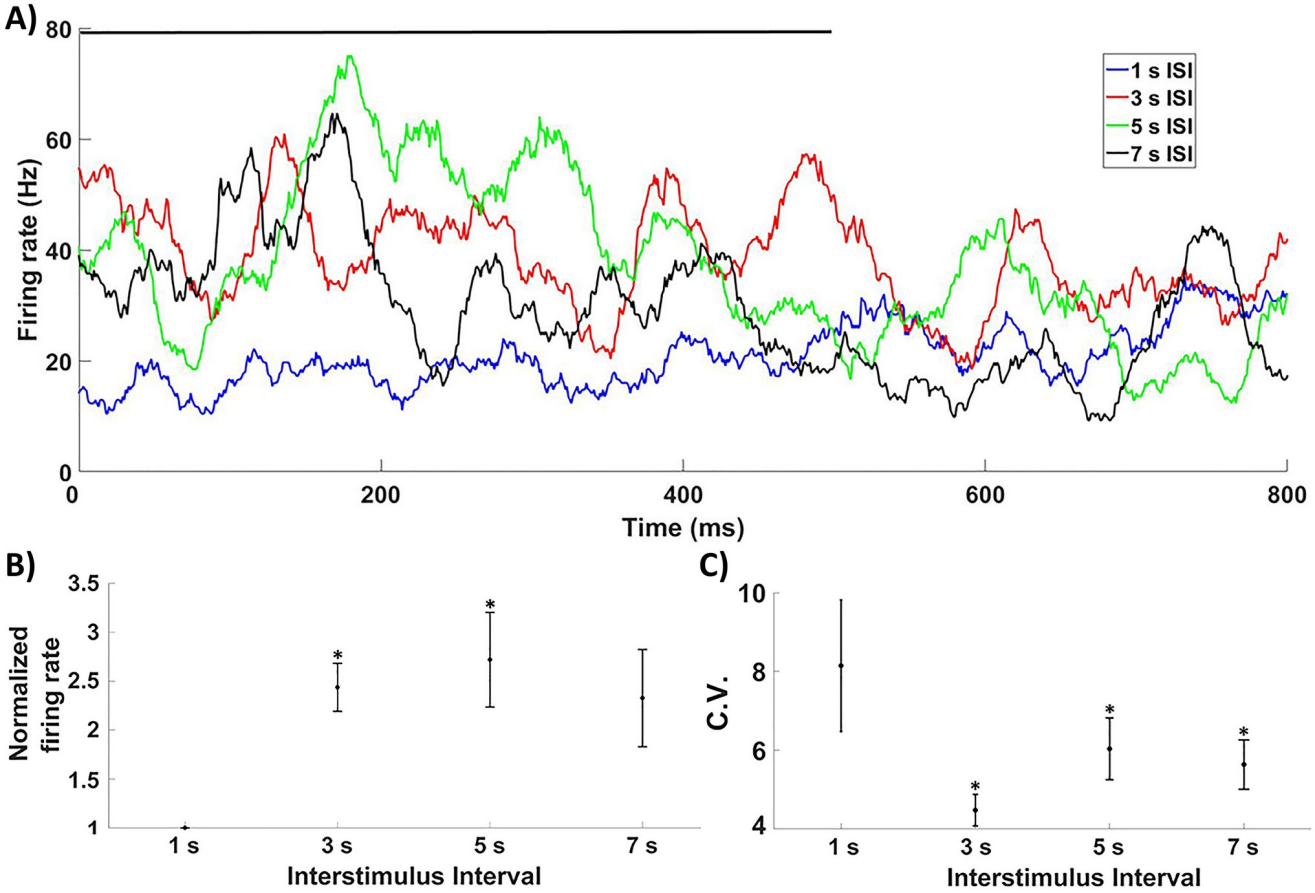
Mean firing rate. A) Mean firing rate dynamics depending on ISI used. Stimulus shown as a black line. B) Normalized mean firing rate during the stimulation window. 3 and 5 s ISIs display significant increases when compared with 1 s ISI. C) C.V. across trials was statistically reduced for longer than 1s ISIs.

Our procedures allow studying whether visual cortex neurons synchronized oscillations do carry information about interstimulus intervals (ISIs) at the level of instant frequency (IF). Fig 2A shows the mean firing rate of all electrodes and presentations during and after stimulation for each recorded ISI. We appreciated an overall increase in the normalized mean firing rate when 3 and 5 seconds ISI were used with respect to 1s ISI (Fig 2B, Wilcoxon test, corrected p value <0.03). These differences were persistent during the whole stimulation window when 5 seconds ISI was used, and restricted to a 300 ms window starting 100 ms after stimulus onset when a 3 second ISI was used (Fig 2A). At last, in the case of 7 versus 1 second ISI, a transient peak of discrimination was found 150–200 ms after stimulus onset (Fig 2A).

We next tried to determine if the responses to visual stimulation with longer ISIs were more stable. As it is known that stimulation quenches neural variability[45], we asked whether changes in the ISI could drive to more or less stable responses for different time lengths. Therefore, we computed the Coefficient of variance (C.V.) during the stimulation window across different presentations in each animal independently to analyze the reliability of the population firing rate in the response to stimulation with different ISI (Fig 2C). We found that C.V. was significantly reduced for ISIs longer than 1s (corrected p value <0.05).

**Fig 3:**
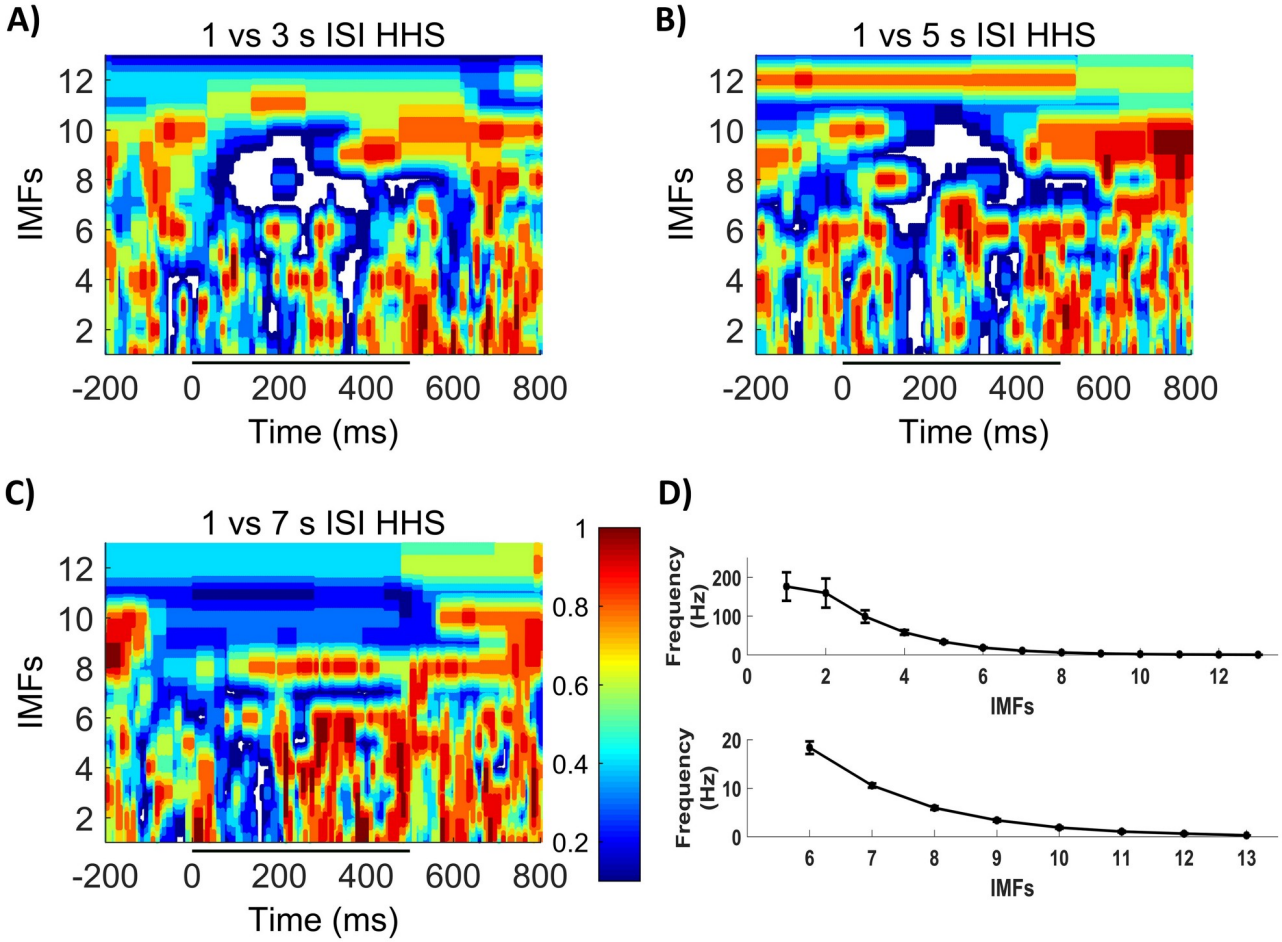
Comparative HHSs IMFs shown in y axis, higher frequencies at the bottom of the HHS. A) P values comparing the obtained HHSs using 1s and 3s ISI. B) P values comparing the obtained HHSs using 1s and 5s ISI. C) P values comparing the obtained HHSs using 1s and 7s ISI. D) Frequency range of each IMF. Wilcoxon P value displayed as color code; unique color bar for the 3 comparisons; significant values displayed in white color. Stimulus displayed as black line.

Then, we studied whether neurons modulated their oscillatory dynamics at the population levels to process ISI length. To do so, we compared the instantaneous amplitude of each IMF across time of 1second ISI with longer intervals (S1 Fig). Thus, we created a “discriminability spectrum” (Fig 3) were the differences in amplitude at each IMF and time point were represented as the p value We appreciated significant differences (white color) in multiple IMFs for 3 and 5 seconds, mainly during and shortly after stimulations (Fig 3A and 3B), but little differences were seen against the 7 seconds ISI (Fig 3C). This suggests that ISI information was encoded simultaneously at different timescales along the response, ranging from [1.87±0.18 to 159.34 ±37.53 Hz], consistent with multiplex theory [36,37].

**Fig 4:**
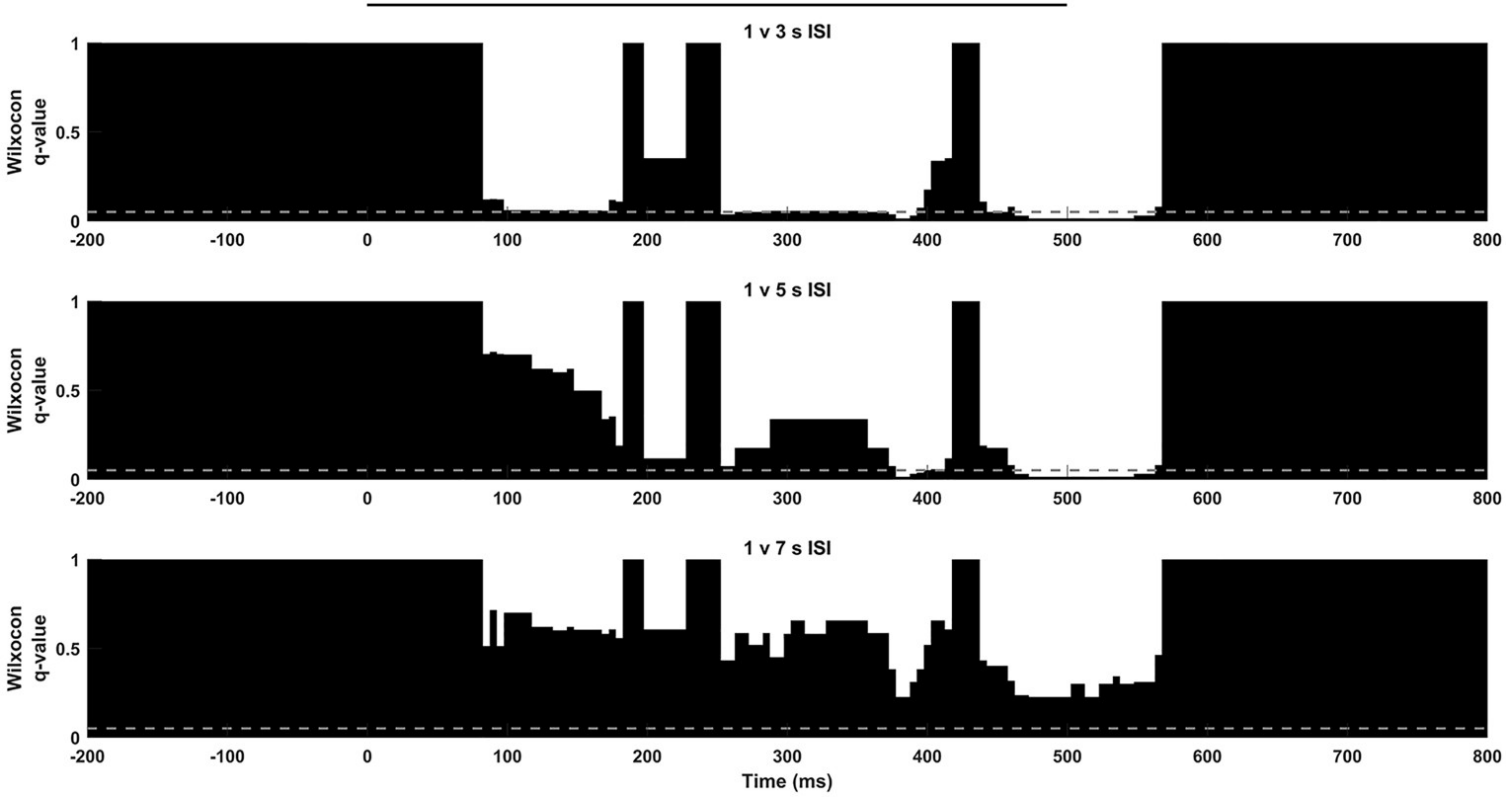
6 Hz population dynamics. IMF 8 (5.93±0.37 Hz). 1s ISI is compared with 3s (top), 5s (middle) and 7 s (bottom) along stimulation using Wilcoxon test and Storey correction. Significance level shown as grey line, stimulus displayed as a black line.

**Fig 5:**
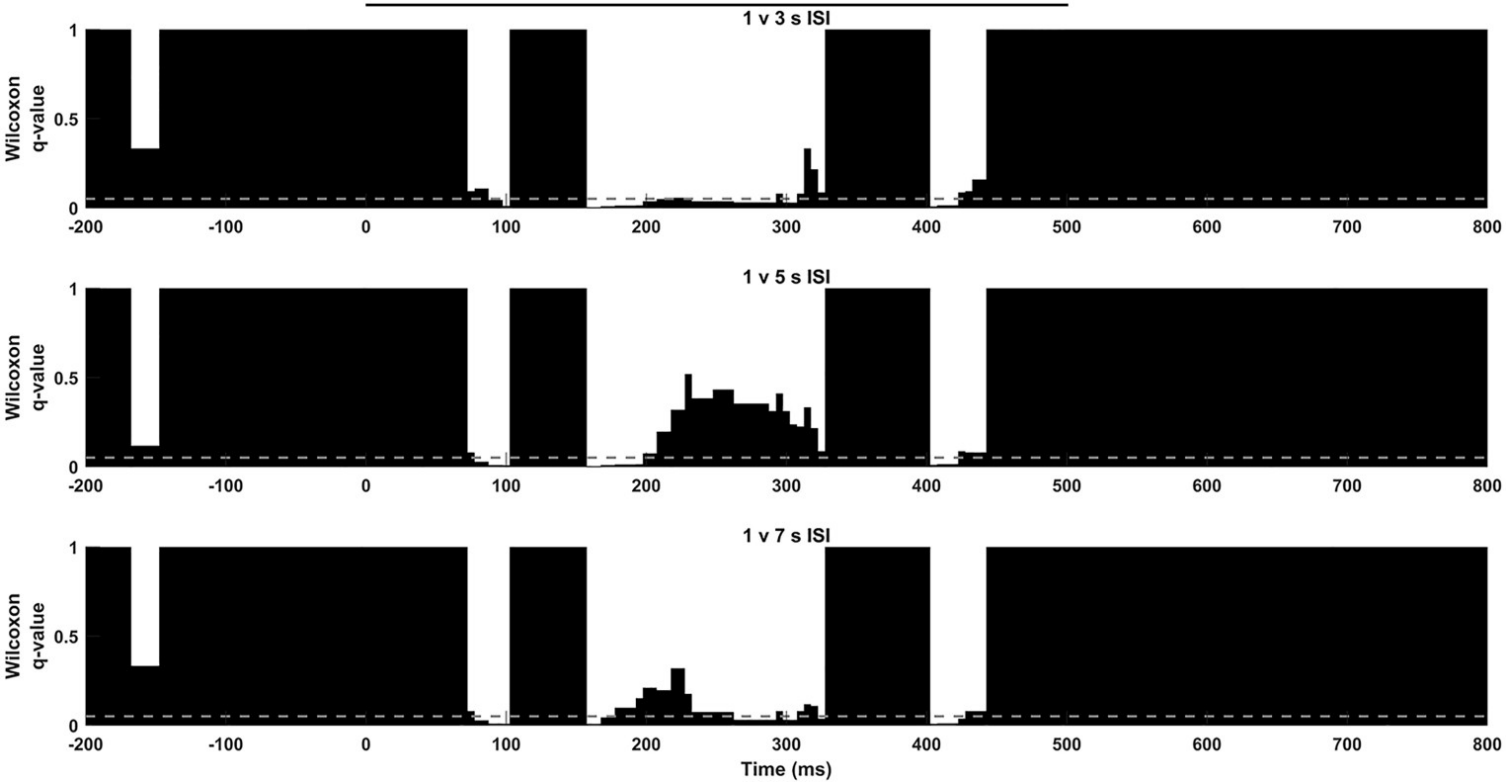
10 Hz population dynamics. IMF 7 (10.56±0.43 Hz). Different temporal discrimination profiles were obtained when 1 s ISI was compared with 3s (top), 5s (middle) or 7 s (bottom). Significance level shown as grey line, stimulus displayed as a black line.

To further study how ISI can be encoded at different frequencies during stimulation we extended our analysis to particular IMFs. We found that IMFs carrying information about low frequencies (Fig 3D), IMFs 7–9 (3.37±0.28 to 10.56±0.43 Hz), were statistically significant or statistically relevant (corrected p value < 0.1) from those at 1 seconds ISI stimulation during almost the whole stimulation window (i.e. IMF 8, Fig 4). Thus, slow frequencies seem to be able to encode information about longer ISIs only until a certain time interval. IMF 8 was of particular interest, since it carried out the responses in the frequency band of the used gratings. We found that this particular phenomenon was reinforced when 3 or 5 seconds ISI were used and was inexistent when we used a 7 seconds ISI. Moreover, the IMF 7 (10.56±0.43 Hz), was also different for each ISI against 1seconds (Fig 5), which suggest that low frequency properties were modified for ISIs longer than 1seconds.

When we extended our analysis to higher frequency components of the response (33.43±2.73 to 159.34±37.53 Hz), the discriminability spectrum became more intermittent (Fig 3A–3C). Statistically significant differences remained present with different temporal profile, especially when 1 second ISI was compared with 3 and 5 seconds intervals. A brief discrimination window emerged ± 200 ms after stimulus onset when 3, 5 and 7 seconds ISI were used against 1 second ISI across several high frequency IMFs, indicating a common high frequency peak in population response for longer ISIs. On the other hand, high frequency components were present in the second half of the stimulation when 3 or 5 seconds ISI were used.

In summary, in our work, we show how the response of neuronal populations of the visual cortex to a grating stimulus is sensitive to the length of the ISI at seconds scale. We described how these differences were present at the level of firing rate, the variability of the response, and in the oscillatory properties of the population response.

## Discussion

Even though the capability of neurons to discriminate temporal patterns is known since the 60’s [46], we are far from understanding how cortical circuits encode time. This statement is particularly true when we refer to times in seconds scale. In this work, we studied how visual cortex neuronal populations of adult rats were responsive to ISI variations in the seconds scale. We found that population firing rate and spiking oscillatory dynamics were sensitive to the ISI we used, suggesting that visual cortex activity encodes temporal information up to several seconds. Furthermore, these range of frequencies were consistent with classical works of neuronal oscillations during visual stimulation [20,21].

The differences were present in the signal amplitude at different frequencies and temporal profiles. Thus we found significant differences at the level of firing rate and low frequency components, including the temporal frequency of the grating. These differences were present throughout the whole stimulating window when 3 or 5 seconds ISI were used and vanished for longer times (7 seconds). Furthermore, there were differences in high frequency components when intervals longer than 1 second were used. These high frequency features appeared 100–120 ms before stimulus offset when 3 or 5 seconds were used, but were not found when 7 seconds ISI were used. In addition, the variability of individual presentations response was reduced when longer than 1-second trails were used, which suggests that longer temporal patterns increase the reliability of the responses. In addition, as it had been previously described [30], the population responses were intermittent and strongly nonlinear, as intrawave modulations were present during the response.

**Fig 6:**
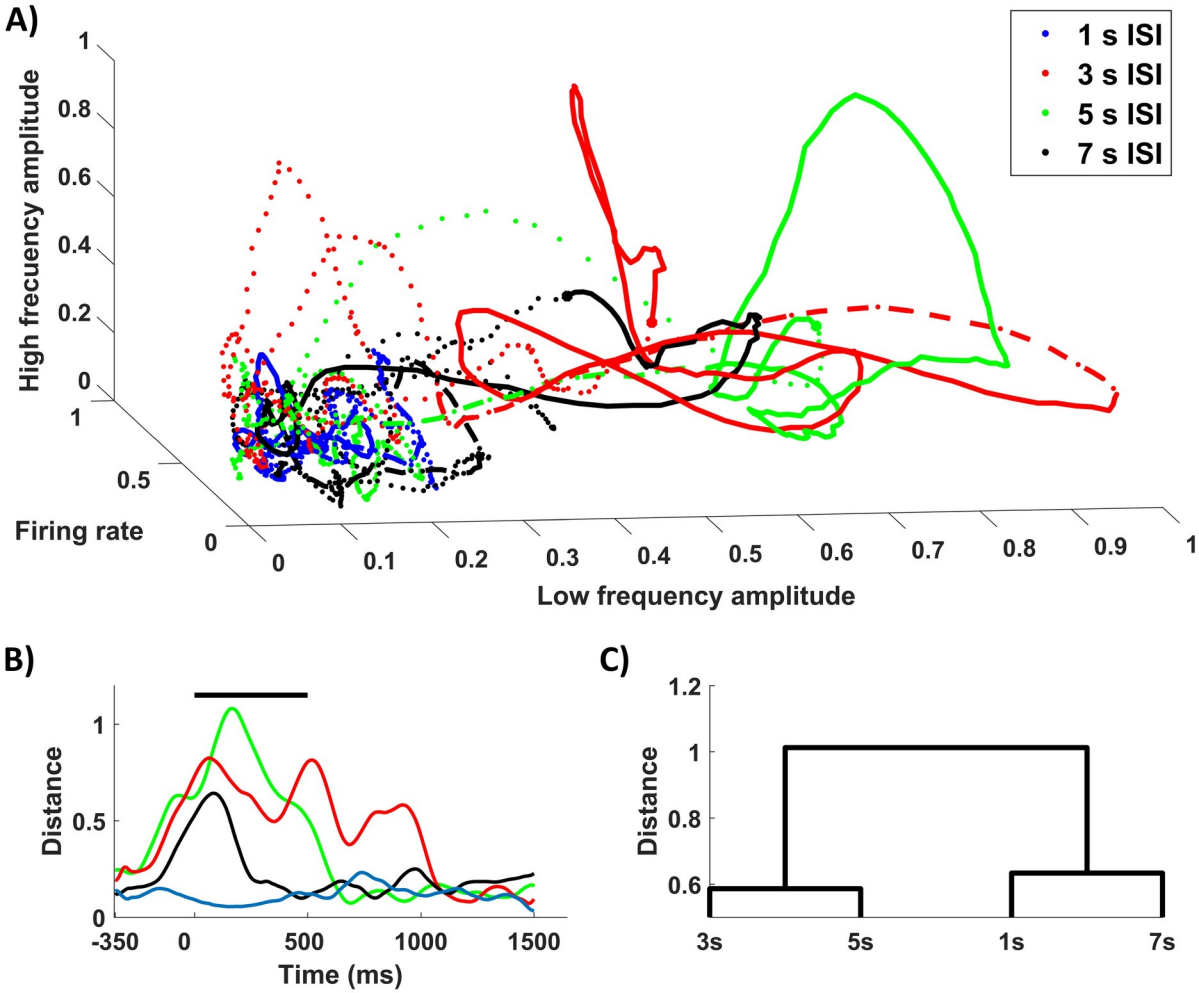
Low dimensional dynamics for ISI discrimination. A) Phase space created using Raw signal mean firing rate, IMF 8 (5.93 ± 0.37 Hz) and IMF 6 (18.37 ± 1.29) as axes where trajectories for all ISIs are plotted ranging from -350 ms to 1500ms after stimulus onset. Trajectories during stimulation shown as whole lines starting as a thick dot during a 300 ms window, starting on stimulus offset as discontinuous line. B) Euclidean distance to the resting state centroid of mean trajectories for the different ISIs. C) Hierarchical tree based on Euclidean distances between trajectories during the stimulation window.

Considering the presented results, we propose a low dimensional phase space for ISI discrimination considering three relevant parameters of the response: firing rate, low frequency (6 Hz) and high frequency (18 Hz) dynamics (Fig 6A), in which visual cortex neuronal populations would be able to discriminate among ISIs depending on the elicited dynamics. In this space, population dynamics are constricted to a fixed point (dotted line, Fig 6A) upon stimulation, when different trajectories are evoked depending on the ISI used during the stimulation. In order to compare the dynamics of the response to different ISIs, we computed the Euclidean distance to the centroid given by the mean point of all trajectories from 400 to 200 ms prior to stimulation (Fig 6B). We can see different temporal dynamics depending on the ISI that we used in this low dimensional space that may lead to ISI discrimination. When 1 second ISI is used, the population trajectory does not move away from the fixed point, as it happens when using longer ISIs. In these series, the evoked trajectories temporal dynamics diverge depending on whether 3/5 seconds ISI or a 7 second ISI was used. In the first case, population dynamics are projected distally to this low-dimensional space during the whole stimulation window. On the other hand, 7 second ISI leads to transient dynamics where the population activity leaves the fixed point for a brief period (±200 ms) and then returns to the previous subspace. Hence, we could easily outline a boundary which would be able to discriminate a certain window of ISIs (3–5 seconds) during stimulation from longer or shorter times. Similar results were seen using IMFs containing oscillations up to 57.98 ± 6.26 Hz in the high frequency axes. These results support the idea of a multiscale response, in which neuronal populations encode ISI information using multiple frequencies and firing rates in their spiking dynamics.

Recent works address the multiscale response in different sensory cortices [47,48], where slow cortical rhythms were proposed to stabilize sensory representation. A different study [49] suggested that different information was carried in the low frequency components of LFP and spikes. Our results show a similar behavior for spiking oscillatory activity in primary visual cortex and support the point of view of multiplexed dynamics for ISI encoding at seconds scale [36,37].

Previous work in the seconds scale on visual cortex was focused on reward timing [12,26]. In these papers, the authors already described a role for oscillatory activity in visual cortex in a different type of timing behavior. Bearing in mind the differences among our study of the interval between visual stimulation and previous work in reward timing, it results interesting that different experiments which involve timing in the seconds scale evoke oscillatory activity in the visual cortex. Nevertheless, oscillations during reward timing were never present before at least 50 correct trials [26], while we describe the oscillatory activity in the response to visual stimulation that was repeated 15 times. Hence, we cannot discuss that the underlying mechanism is the same for the oscillations in the activity of visual cortex that are modulated by the interval in visual stimulation and reward timing.

Given that temporal coding at seconds scale has been reported to be crucial for behavior [50,51], the presence of an optimal temporal window of response to stimuli is an important question that should be assessed in future studies (Fig 6A). Our results suggest that neuronal population response dynamics strongly differ when 1 vs 3 seconds and 5 second ISI are used for a vertical grating stimulus (Fig 6C). These differences are softened when a longer ISI (7 seconds) was used. Therefore, we may consider facilitation at certain frequencies for intermediate times in the temporal scale of seconds. No clear differences between 3 and 5 seconds ISIs were seen during stimulation. Thus, it might reflect a window of time in which visual cortex response to stimulation is strengthened.

An important question remaining to answer is the source of these oscillatory dynamics. Although in this work we describe temporal discrimination in visual cortex, this kind of computational studies has been classically studied in prefrontal cortex [52–54] but, at present, it is still unclear how prefrontal and sensory cortices interact during interval timing at seconds scales. Future studies will be required to assess the communication and hierarchical organization of these areas during the estimation of interval time, as well as the probable effect of dopamine via the frontostriatal circuit [13,55].

## Supporting information

S1 Fig Representative examples of individual stimulation. Evoked population response to single stimulation using A) 1s ISI B) 3s ISI C) 5s ISI D) 7s ISI. Top, PSTH summing the activity in all the electrodes in response to stimulation; Bottom, HHS spectrum of the signal above. Stimulus displayed as a black line.
(TIF)
