# Supplementary figures and images for: Multiscale dynamics of interstimulus interval integration in visual cortex
Source: PLoS One. 2018 Dec 17;13(12):e0208822. doi: 10.1371/journal.pone.0208822 (PMC6296521; doi:10.1371/journal.pone.0208822)

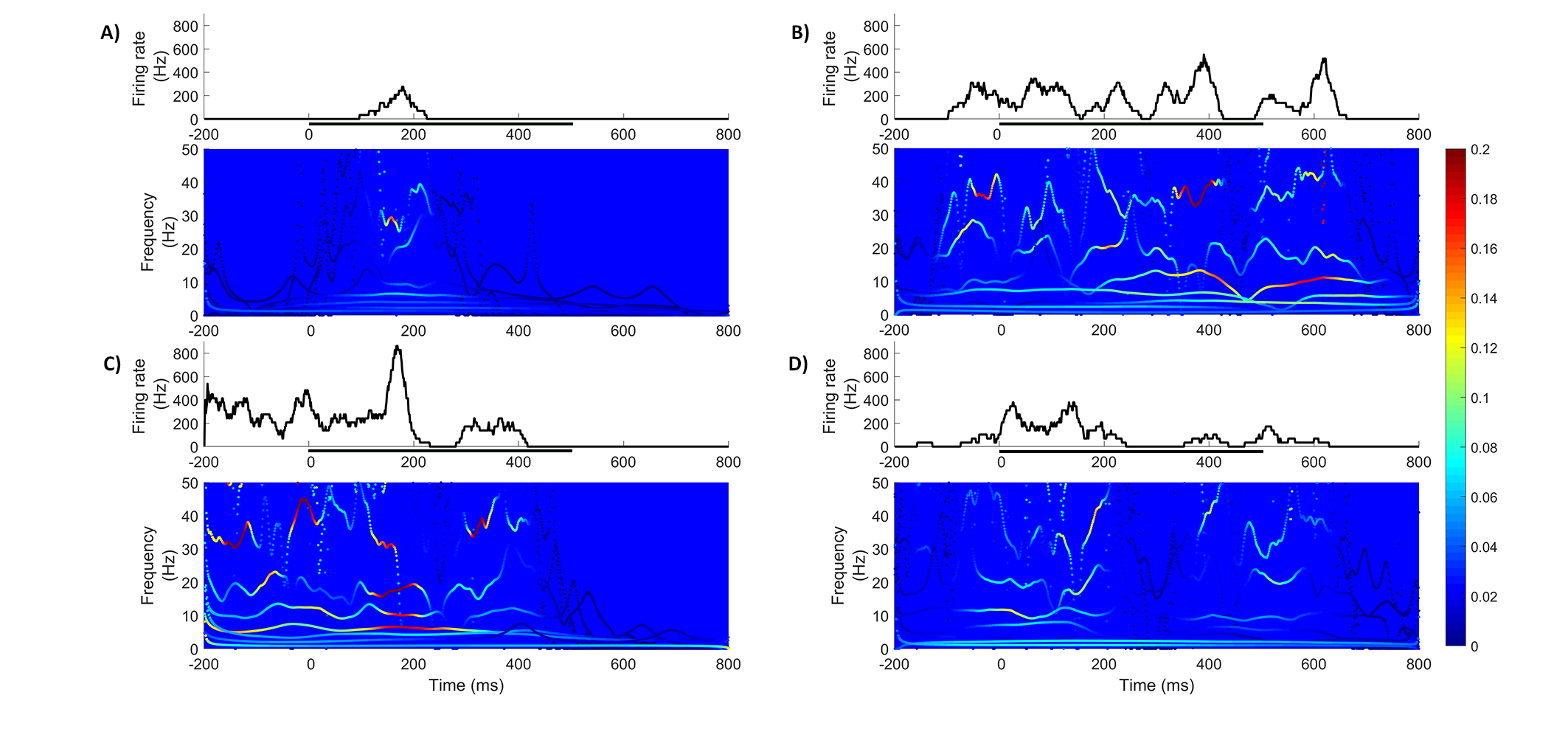

Supplement: S1 Fig — Evoked population response to single stimulation using A) 1s ISI B) 3s ISI C) 5s ISI D) 7s ISI. Top, PSTH summing the activity in all the electrodes in response to stimulation; Bottom, HHS spectrum of the signal above. Stimulus displayed as a black line. (TIF) [file pone.0208822.s001.tif]
